# Dietary sodium butyrate improves female broiler breeder performance and offspring immune function by enhancing maternal intestinal barrier and microbiota

**DOI:** 10.1016/j.psj.2023.102658

**Published:** 2023-03-18

**Authors:** Chuanpi Xiao, Li Zhang, Bo Zhang, Linglian Kong, Xue Pan, Tim Goossens, Zhigang Song

**Affiliations:** ⁎ Key Laboratory of Efficient Utilization of Non-grain Feed Resources, Department of Animal Science, Shandong Agricultural University, Taian, Shandong, China; † Precision Livestock and Nutrition Unit, Gembloux Agro-Bio Tech, University of Liège, Gembloux, Belgium; ‡ Adisseo Life Science Products (Shanghai) Co. Ltd., Shanghai, China; § Adisseo France, Hoogveld, Dendermonde, Belgium

**Keywords:** broiler breeder, gut microbiota, offspring, reproductive performance, sodium butyrate

## Abstract

This study aimed to investigate the effects of dietary sodium butyrate (**SB**) supplementation on the reproductive performance of female broiler breeders under intensive rearing conditions and to analyze antioxidant capacity, immune function, and intestinal barrier function of the female breeders and their offspring. A total of 96,000 40-wk-old Ross308 female broiler breeders were divided into the control (**CON**) and SB groups, each with 6 replicates of 8,000 birds. Each house with similar production performance characteristics was considered a replicate. The experiment lasted for 20 wk, whereupon sampling took place. Results showed that SB improved the egg production performance, egg quality of broiler breeders, and hatchability (*P* < 0.05). Maternal supplementation with SB substantially increased serum immunoglobulin A levels in broiler breeders and offspring (both *P* = 0.04) and offspring immunoglobulin G (*P* < 0.001). The levels of interleukin-1β (*P* < 0.001) and interleukin-4 (*P* = 0.03) in the offspring were downregulated, while the total superoxide dismutase in the offspring and the eggs increased (*P* < 0.05). The serum biochemical components in breeders and offspring were altered by SB, as evidenced by the reduction in triglycerides, total cholesterol, and high- and low-density lipoproteins (*P* < 0.05). The intestinal morphology of broiler breeders and offspring also improved by the SB with the decreasing the jejunal crypt depth (*P* = 0.04) and increasing villus height in offspring (*P* = 0.03). Maternal jejunal and ileal intestinal barrier-related genes were also shown to be significantly affected by SB. Furthermore, SB altered the microbial diversity in maternal cecal contents, thus increasing the abundance of *Lachnospiraceae* (*P* = 0.004) and *Ruminococcaceae* (*P* = 0.03). Dietary SB enhanced the reproductive performance and egg quality of broiler breeders and improved the antioxidant capacity and immune function of broiler breeders and offspring, with the benefits potentially arising from the regulation of the maternal intestinal barrier and gut microbiota by SB.

## INTRODUCTION

The emergence and spread of antibiotic resistance in pathogens has led to restrictions in some countries on antibiotic growth promoters in animal feed (Huang et al., 2018). Alternatives to the antibiotic growth promoters and regulation of immune function in livestock have attracted global interest in the field of animal nutrition (Kim et al., 2021). Some natural extracts or chemical products, such as enzymes, probiotics, organic acids, and plant extracts, play an increasingly important role as alternatives to antibiotics for the prevention of diseases and the promotion of animal growth (Zhu et al., 2021).

Butyric acid is a short-chain fatty acid (**SCFA**) produced in the hindgut segment by the enzymatic digestion of carbohydrates and proteins by probiotic bacteria (Luu et al., 2019). After being absorbed through the intestine, it is transported through the portal vein to the liver before being synthesized into acetyl coenzyme A and metabolized into ketone bodies, which can provide direct energy to the intestinal mucosal cells after further synthesis of adenine nucleoside triphosphate (Xia et al., 2017; Gabbianelli et al., 2020). Butyric acid has the ability to provide intestinal epithelial cells with natural energy for cell proliferation and damage repair (Fellows et al., 2018). Sodium butyrate (**SB**) has accordingly been used as a green additive instead of antibiotics to improve growth performance and intestinal health (Zou et al., 2019; Wang et al., 2021b). Dietary SB results in improved performance by promoting gastrointestinal development in poultry and has a dose-related effect (Lan et al., 2020). In laying hens, SB influences nutrient digestibility, thereby affecting production performance (Zhang et al., 2022a), as well as amino acid metabolism and intestinal immune function induced by cecal microorganisms (Zhong et al., 2022). In the modern broiler industry, where rapid growth rate is often associated with increased stress and immune challenges, SB can modulate the intestinal permeability of broilers and regulate the intestinal microbial community (Naghizadeh et al., 2022), thereby improving immune function and stress resistance (Bortoluzzi et al., 2017).

Considering the long service life and significance of broiler breeders in establishing the poultry industry chain, they are prone to transmitting zoonotic bacteria, such as *E. coli* and *Salmonella*, through the food chain (Magdy et al., 2020). In terms of antibiotic reduction and alternative applications, better measures are required to limit the spread of harmful bacteria (Kim et al., 2022). Therefore, utilizing feed such as organic acids or plant extracts to enhance the immune function and intestinal health of broiler breeders becomes a necessary intervention (Swaggerty et al., 2022). The primary immune function of the offspring is related to the immune status of the maternal hen, and enhancing the immunity of the offspring may result in economic benefits (Berghof et al., 2013). However, systematic research has not been conducted into the effects of SB on the immune status and intestinal health of broiler breeders and offspring, and limited studies have focused on the association between nutrition and intestinal health of broiler breeders in intensive rearing farms. The present study aimed to investigate the effect of dietary SB on serum biochemical indices, lipid metabolism, and the intestinal microbial community of broiler breeders and offspring. It also aimed to reveal the theoretical basis behind the use of SB to improve the reproductive performance of broiler breeders and the antioxidant capacity, immune function, and intestinal health of female breeders and their offspring.

## MATERIALS AND METHODS

### Experimental Design and Animal Management

All experimental procedures were approved by the Ethics Committee of Shandong Agricultural University and performed in accordance with the Guidelines for Experimental Animals of the Ministry of Science and Technology (Beijing, China). Furthermore, all feeding and euthanasia procedures were carried out with the welfare of the animals in mind.

A total of 96,000 forty-wk-old ROSS 308 female broiler breeders were randomly divided into the CON (basal diet) and SB group (basal diet supplemented with 1,000 mg/kg sodium butyrate). Each treatment group consisted of 6 replicates, one for each house with the same age and similar production performance, with 8,000 female hens in each house. The purity of SB was 30%, and it was supplied by Adisseo Life Science Products (Shanghai, China). The additive amount of SB was recommended by the manufacturer. The breeding experiment was conducted at the commercial farm of Shandong Hekangyuan Group (Liaocheng, China). The basal diet was a pelleted diet, and the feed and nutrient compositions are shown in Supplementary Table S1.

In each house, 8,000 female and 900 male breeders were mixed together for spontaneous mating at a stocking density of approximately 5 birds/m^2^. All birds were housed in a 2/3 slatted floor system, which included an elevated floor of wooden slats, and the remaining area was covered with wood shavings on the ground as litter. The height of the feeding trough was adjusted, and restriction devices were used to ensure that both males and females received separate feed. Each female was provided with a limited amount of feed (130 g in total) at 8 am and 2 pm daily to avoid excessive weight gain and to allow free access to water. The males did not receive any dietary treatments and were fed with 150 g of feed per day. Broiler breeders were housed in an ideal environmentally controlled room using 16 h/d of light at an ambient temperature of 20°C. Eggs were collected daily after feeding and placed in a light-protected room at an ambient temperature of 10°C until hatching. The entire experimental period lasted for 20 wk.

### Sample Collection

Twelve broiler breeders per treatment (2 per replicate) with similar body weights were randomly selected for sample collection after production data were recorded during the entire period. The blood was extracted from the broiler wing vein using a sterile syringe. Approximately 3 mL of blood was transferred to a tube without anticoagulant, placed for 30 min, and then centrifuged for 10 min with 1,000 × *g* to separate the upper liquid layer for serum, which was stored in a −20°C freezer for further analysis. Segments (2 cm) of the intestine from the jejunum and ileum were dissected and gently rinsed with cold saline before immersing in 10% formalin solution for histological analysis. Approximately 2 g of liver, jejunum, and ileum tissue samples from broiler breeders were collected in the same locations, and then rapidly frozen in liquid nitrogen, and transferred to a freezer at −80°C for further testing. Serum samples were collected from chicks within 12 h after hatching following the same procedure described for broiler breeders. Liver, jejunum, and ileum samples from triplicate numbers of chicks were also obtained and stored. Three samples from the same replicate were combined into one sample for examination to ensure the sufficiency of sampling. The egg whites and yolks of the breeder eggs were separated and placed in a freezer at −80°C for the next step of the analysis. Samples of broiler breeder cecum contents were collected on ice and immediately transferred to a freezer at −80°C for 16S rRNA analysis.

### Egg Production Capacity, Egg Quality, and Hatching Performance

During the experiment, egg production, egg weight, and the number of substandard eggs were recorded daily. Unqualified eggs included misshapen eggs, dirty eggs, broken eggs, or eggs with incomplete shell development. The laying rate, average egg weight, qualified egg rate, and feed conversion rate were calculated on a replicate basis.

During the last week, the 120 eggs collected for egg quality determination consisted of 10 eggs from each replicate. Eggshell thickness was measured using an ETG-1061 type instrument (Robotmation Corporation, Minato City, Japan). The strength of the eggshell was examined using an eggshell light measuring device (EFG-0.5.3 type, Robotmation Corporation). A multifunctional egg quality detector was used to measure egg white height, yolk color, and Hastelloy unit (ETM-5200, Robotmation Corporation). The yolks were separated using a separator and weighed. The horizontal and vertical diameters of the eggs were determined using electronic vernier calipers.

All eggs during the experimental period were placed in the farm's own Yiai-19200 automatic incubator and incubated according to standardized procedures with a temperature of 38.3°C from d 1 to 5, 38.1°C from d 6 to 12 and 37.9°C for d 13 to 19. At hatching, a temperature of 37.2°C was maintained. Relative humidity was at 60% for d 1 to 7, 50% for d 8 to 18 and 70% for d 19 to 21. On the eighth day of incubation, unfertilized eggs were removed after lighting, and the number of unfertilized eggs was recorded for each replicate. After incubation, the number of chicks, their weight, and the number of healthy chicks were recorded. The fertilization rate, hatching rate of fertilized eggs, and percentage of healthy chicks were calculated.

### Analysis of Serum Biochemical Components

Glucose, triglyceride (**TG**), total cholesterol (**TCHO**), high-density lipoprotein cholesterol (**HDL**), and low-density lipoprotein cholesterol (**LDL**) levels in serum samples from broiler breeders and chicks were measured using a fully automated biochemical analyzer (7020; Hitachi, Co., Ltd., Tokyo, Japan).

### Fat-Soluble Vitamin Assay

Fat-soluble vitamins, including vitamin A, D, E, and K, were measured in the serum of broiler breeders, chicks, and egg yolk by using the kits obtained from Nanjing Jiancheng Institute of Biotechnology (Nanjing, China). All assay procedures were performed in strict accordance with the manufacturer's instructions. The intrabatch variability index of the data was less than 5%, and the interbatch variability index was less than 8%.

### Analysis of Immune Indicators and Antioxidant Status

Serum interleukin-1β (**IL-1β**), interleukin-4 (**IL-4**), interleukin-6 (**IL-6**), endotoxin, immunoglobulin A (**IgA**), and immunoglobulin G (**IgG**) levels of the female breeders l and offspring were assayed using enzyme-linked immunosorbent assay (**ELISA**) kits (MLBIO Co., Shanghai, China).

Approximately 0.2 g of liver and egg albumen samples were homogenized in phosphate-buffered saline, and the supernatant was collected after centrifugation. Total superoxide dismutase (**T-SOD**) was determined in maternal and offspring serum and liver by using the kits provided by Nanjing Jiancheng Institute of Biotechnology (Nanjing, China). T-SOD in egg albumen was also detected. Jejunum samples from broiler breeders were homogenized using the above method and assayed for secretory immunoglobulins using an ELISA kit (MLBIO Co., Shanghai, China) to detect the content of secretory immunoglobulin A (**sIgA**). All assay procedures were performed in strict accordance with the manufacturer's instructions. The intrabatch variability index of the data was less than 5%, and the interbatch variability index was less than 8%.

### Jejunum Morphology Analysis

Jejunum samples from broiler breeders and offspring were fixed in a 10% formalin solution for 24 h. Tissues were embedded in paraffin and stained with hematoxylin and eosin by slicing the 5 µm-thick tissues onto the slides by using a microtome. The jejunal sections were observed and photographed using a Nikon 80i (Tokyo, Japan) microscope. The intestinal morphology was analyzed using Image J software. Villus height (**VH**) is the distance from the tip of the villus to the bottom crypt junction, while crypt depth (**CD**) is the depth of the depression between 2 adjacent villi. Ten representative data per sample were selected for statistical analysis.

### RNA Extraction and Real-Time PCR

Total RNA was extracted from maternal and progeny jejunum and ileum with an RNA rapid isolation kit (Vazyme Biotech, Nanjing, China) according to the manufacturer's instructions. The purity of RNA samples was determined using a NanoDrop spectrophotometer (ND-2000, Thermo Scientific, Wilmington, NC). The RNA integrity was detected via 1% agarose gel electrophoresis. Reverse transcription of 1 μg of total RNA was conducted using a PrimeScript RT reagent kit (RR047A, TaKaRa, Japan). Gene expression was assayed by RT-PCR analysis by using TB Green Premix Ex Taq (RR820A, Takara, Japan) on an ABI 7500 real-time PCR system (Thermo Scientific, Wilmington, NC) as previously described (Xiao et al., 2022). The primer sequences are shown in Supplementary Table S2. The amplification efficiency of the primers was verified using standard curves, while the specificity of the amplified products was verified using melting curves. The geometric means of *β-actin* and glyceraldehyde-3-phosphate dehydrogenase (***GADPH***) gene expression were used to normalize the results. The relative quantification of each gene expression was calculated using the 2^−ΔΔCT^ method.

### 16S rRNA Gene Amplification Sequencing and Analysis

Total genomic DNA was extracted from cecal contents using the EZNA soil DNA kit (Omega Bio-Tek, Norcross, CA) The extracted DNA was analyzed using a NanoDrop spectrophotometer (ND-2000, Thermo Scientific, Wilmington, NC). The integrity of the DNA was determined by 1% agarose gel electrophoresis, followed by PCR amplification of the V3 to V4 variable region of the bacterial 16S rRNA gene using specific primers 338F and 806R (GeneAmp9700 produced by ABI). The amplification procedure involved a 3 min reaction at 95°C, followed by 27 cycles (95°C for 30 s, annealing at 55°C for 30 s, 72°C for 30 s), and a final step of extension at 72°C for 10 min. PCR was performed in a 20 μ L mixed system containing 10 ng of template DNA. PCR products were recovered on 2% agarose gel, purified using the AxyPrep DNA gel extraction kit (Axygen Biosciences, Union City, CA), and then quantified using QuantiFluor-ST (Promega, Madison, WI).

Purified amplification products were mixed in equimolar amounts, and paired-end sequenced (2 × 300) on the Illumina MiSeq PE300 platform (Illumina, San Diego, CA, USA). The raw sequences were quality-screened with Quantitative Insights Into Microbial Ecology 2 (**QIIME2**) software, filtered, trimmed, denoised, and merged with demultiplexed sequences for each sample. Next, the chimeric sequences were identified and removed to obtain a table of amplicon sequence variation characteristics. The database obtained in the previous step was pruned to the V3 to V4 region according to the 338F/806R primers to bring the species classification table. After removing all contaminated sequences, bacteria with abundant differences between samples and groups were identified using ANCOM, ANOVA, Kruskal-Wallis test, and LEFSE. The horizontal alpha diversity indices of the characteristic sequences were calculated using QIIME2 core diversity indices for estimating microbial diversity of individual samples, including indices such as observed operational taxonomic units (**OTUS**), Chao1 abundance estimator, Shannon diversity index, and Faith phylogenetic diversity index. The beta diversity index included the Bray-Curtis index, unweighted UniFrac index, and weighted UniFrac index, which were used to evaluate the variation in microbial community structure between samples and could be visualized by principal coordinate analysis (**PCoA**).

### Statistical Analysis

Data were presented as mean ± SD. All data were calculated and analyzed on a replicate basis. Differences between treatments were analyzed using *t* test in SPSS 22.0 (SPSS. Inc., Chicago, IL). Statistical significance was considered at *P* < 0.05.

## RESULTS

### Laying and Hatching Performance

No significant difference was observed in the average egg weight and percentage of qualified eggs between these 2 diet treatment groups (Table 1). However, in comparison with the control group, SB significantly increased the laying rate (*P* = 0.02) and decreased the feed conversion ratio (*P* < 0.001). In addition, birds in the SB treatment group exhibited better hatching performance, as reflected by increased hatching and healthy chick rates. By contrast, larger chick weights were observed (*P* < 0.001).

**Table 1:** Effect of dietary sodium butyrate on the laying and hatching performance of broiler breeders.

Parameter	Treatment		*P* value
	CON	SB	
Laying rate, %	66.37±0.87^b^	70.18±0.90^a^	0.02
Average egg weight, g	71.31±0.69	70.71±1.32	0.69
Percentage of qualified eggs, %	98.37±0.17	98.44±0.12	0.75
Feed conversion ratio, g/g	2.80±0.01^a^	2.69±0.02^b^	<0.001
Fertility, %	91.34±0.08^b^	92.38±0.18^a^	<0.001
Percentage of fertile eggs hatched, %	96.30±0.12	97.23±0.07	0.06
Healthy chick rate, %	87.24±0.35^b^	88.63±0.07^a^	<0.001
Chick weight, g	46.04±0.64^b^	49.16±0.53^a^	<0.001

Values are expressed as the mean ± SD (*n* = 6).

Different superscripts (a, b) in the same line indicate significant differences (*P* < 0.05).

Abbreviations: CON, control, birds fed a basal diet; SB, sodium butyrate, birds fed a basal diet containing 300 mg/kg SB.

### Egg Quality

Table 2 presents the effects of SB dietary treatment on egg quality indices. The eggshell strength was significantly increased in the SB treatment group compared with the control group (*P* < 0.001). However, SB did not affect eggshell thickness, eggshell weight, egg shape index, protein height, and Haugh units. By contrast, eggshell color was significantly reduced in the SB treatment group (*P* = 0.03).

**Table 2:** Effect of dietary sodium butyrate on the egg quality of broiler breeders.

Parameter	Treatment		*P* value
	CON	SB	
Eggshell thickness, mm	3.02±0.04	2.97±0.06	0.52
Eggshell strength, N	35.97±1.15^b^	42.31±1.33^a^	<0.001
Eggshell weight, g	6.72±0.07	6.85±0.10	0.31
Egg shape index, mm/mm	1.33±0.01	1.32±0.01	0.47
Albumen height, mm	6.61±0.24	6.76±0.17	0.62
Haugh unit	77.25±1.94	78.61±1.13	0.55
Yolk color	7.81±0.19^a^	6.86±0.36^b^	0.03

Values are expressed as the mean ± SD (*n* = 6).

Different superscripts (a, b) in the same line indicate significant differences (*P* < 0.05).

Abbreviations: CON, control, birds fed a basal diet; SB, sodium butyrate, birds fed a basal diet containing 300 mg/kg SB.

### Serum Biochemical Indexes

Table 3 summarizes the effects of the experimental dietary treatments on the serum biochemical components of the breeders and offspring. The levels of TG, TCHO, HDL, and LDL were reduced by SB supplementation in the serum of breeder and offspring (*P* < 0.05).

**Table 3:** Effect of dietary sodium butyrate on the serum biochemical indexes of broiler breeders.

Parameter	CON	SB	*P* value
Breeders			
GLU, mmol/L	13.09±0.29	13.48±0.20	0.29
TG, mmol/L	16.64±1.29^a^	12.27±0.95^b^	0.02
TCHO, mmol/L	5.00±0.16^a^	3.68±0.15^b^	<0.001
HDL, mmol/L	2.03±0.05^a^	1.63±0.07^b^	<0.001
LDL, mmol/L	1.30±0.06^a^	0.97±0.05^b^	<0.001
Offspring			
GLU, mmol/L	11.88±0.22	12.28±0.32	0.35
TG, mmol/L	0.70±0.03^a^	0.57±0.03^b^	0.02
TCHO, mmol/L	12.01±0.41^a^	9.39±0.42^b^	<0.001
HDL, mmol/L	4.80±0.13^a^	4.28±0.18^b^	0.04
LDL, mmol/L	3.21±0.13^a^	2.49±0.19^b^	0.01

Values are expressed as the mean ± SD (*n* = 6).

Different superscripts (a, b) in the same line indicate significant differences (*P* < 0.05).

Abbreviations: CON, control, birds fed a basal diet; SB, sodium butyrate, birds fed a basal diet containing 300 mg/kg SB.

### Fat-Soluble Vitamin Contents

**Figure 1:**
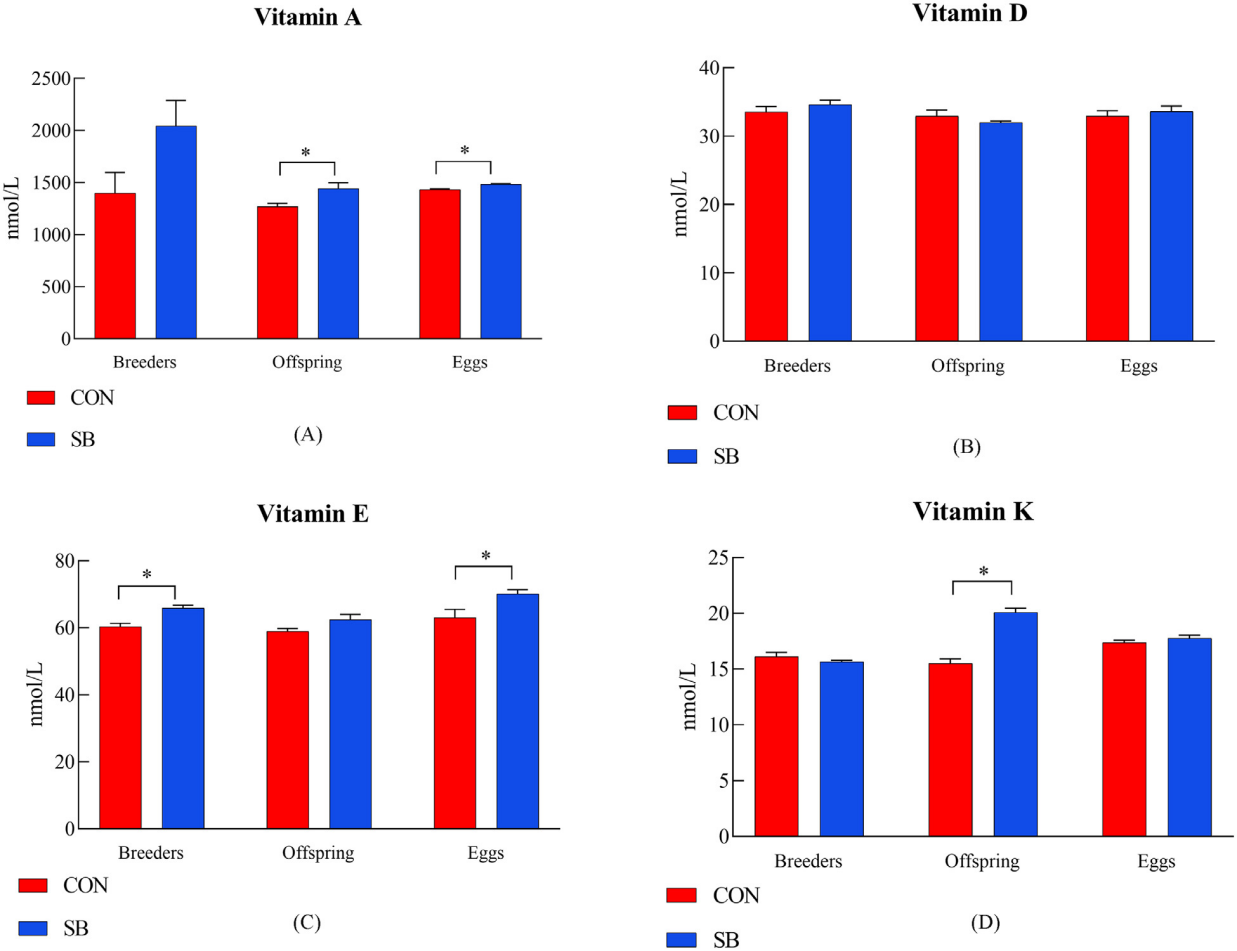
Effect of dietary sodium butyrate on the fat-soluble vitamins of broiler breeders, offspring, and eggs. (A) Vitamin A content in the serum of broiler breeders and offspring and in the egg yolk; (B) Vitamin D content in the serum of broiler breeders and offspring, and egg yolk; (C) Vitamin E content in the serum of broiler breeders and offspring, and egg yolk; (D) Vitamin K content in the serum of broiler breeders and offspring and in the egg yolk. *P < 0.05, compared with another group. Abbreviations: CON, control, birds fed a basal diet; SB, sodium butyrate, birds fed a basal diet containing 300 mg/kg SB.

As shown in Figure 1, the contents of fat-soluble vitamins were remarkably different among breeder, egg and offspring serum. The level of vitamin E in the serum of breeders was substantially increased with SB supplementation (*P* < 0.001). The concentrations of vitamin A and K in the serum of offspring and vitamin A and E in the eggs were significantly higher (*P* < 0.05) than the controls.

### Antioxidation and Immune Functions

The activity of T-SOD in the liver of the offspring of the SB group was significantly greater than that of the CON group (*P* = 0.02), and the chick serum showed the same trend (*P* < 0.001, Table 4). The T-SOD activity in the eggs was upregulated by SB. No differences were observed in the IL-6 and endotoxin concentrations of the serum between breeders compared with the offspring of these 2 groups (Table 5). The experimental treatment increased IgA in the serum of the breeder and offspring and IgG in the offspring. The offspring showed in contrast a significant decrease in IL-1β and IL-4 in the serum (*P* < 0.05).

**Table 4:** Effect of dietary sodium butyrate on the T-SOD of broiler breeders, eggs, and offspring.

Parameter	CON	SB	*P* value
Breeders			
Liver, pg/mg prot	694.65±19.08	778.54±53.11	0.19
Serum, pg/mL	1610.43±80.39	1750.68±95.21	0.20
Offspring			
Liver, pg/mg prot	1049.20±89.26^b^	1320.22±40.00^a^	0.02
Serum, pg/mL	497.59±28.67^b^	640.15±21.79^a^	<0.001
Eggs			
Egg, pg/mg prot	55.32±1.25^b^	63.07±1.72^a^	<0.001

Values are expressed as the mean ± SD (*n* = 6).

Different superscripts (a, b) in the same line indicate significant differences (*P* < 0.05).

Abbreviations: CON, control, birds fed a basal diet; SB, sodium butyrate, birds fed a basal diet containing 300 mg/kg SB; T-SOD, total superoxide dismutase.

**Table 5:** Effect of dietary sodium butyrate on the serum immune function of broiler breeders and offspring.

Parameter	CON	SB	*P* value
Breeders			
IL-1β, pg/mL	354.74±8.87	341.15±12.34	0.40
IL-4, pg/mL	146.78±5.13	143.98±4.15	0.68
IL-6, pg/mL	25.18±0.62	25.05±0.50	0.87
Endotoxin, pg/mL	73.52±1.69	69.76±0.64	0.07
IgA, pg/mL	230.02±5.46^b^	248.90±6.64^a^	0.04
IgG, pg/mL	2268.43±90.81	2267.83±36.09	0.99
Offspring			
IL-1β, pg/mL	400.44±0.72^b^	413.85±2.03^a^	<0.001
IL-4, pg/mL	155.26±1.76^b^	162.67±2.04^a^	0.03
IL-6, pg/mL	30.03±0.48	29.75±1.17	0.83
Endotoxin, pg/mL	86.15±3.41	83.44±1.97	0.48
IgA, pg/mL	258.52±8.50^b^	293.08±12.33^a^	0.04
IgG, pg/mL	2542.63±58.18^b^	2871.38±77.19^a^	<0.001

Values are expressed as the mean ± SD (*n* = 6).

Different superscripts (a, b) in the same line indicate significant differences (*P* < 0.05).

Abbreviations: CON, control, birds fed a basal diet; IgA, immunoglobulin A; IgG, immunoglobulin G; IL-1β, interleukin 1β; IL-4, interleukin 4; IL-6, interleukin 6; SB, sodium butyrate, birds fed a basal diet containing 300 mg/kg SB.

### Intestinal Morphology and Barrier Function Gene Expression

**Figure 2:**
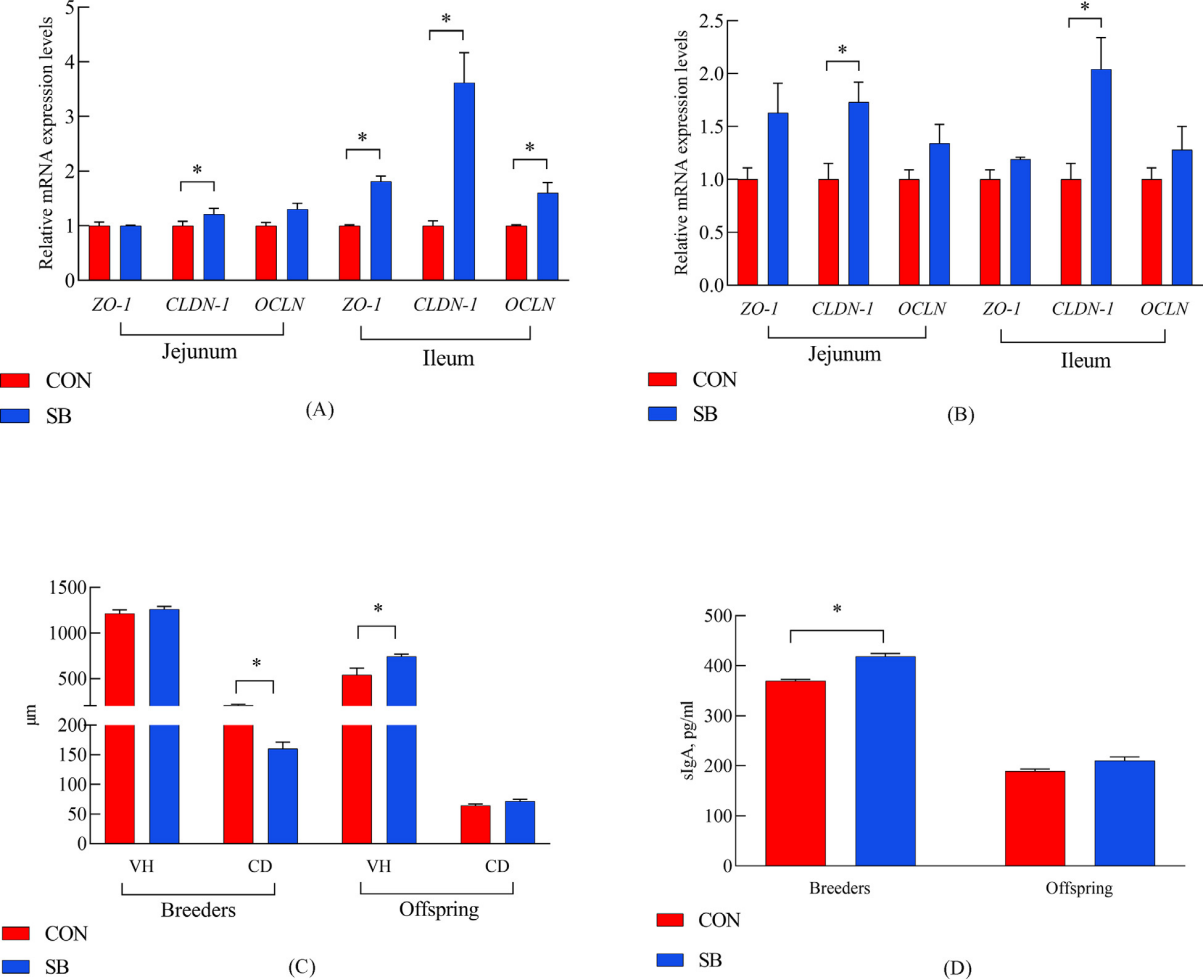
Effects of dietary sodium butyrate on the intestinal barrier and histomorphology of broiler breeders and offspring. (A) Jejunal and ileal barrier gene expression in broiler breeders; (B) Jejunal and ileal barrier gene expression in the offspring; (C) Histomorphology of the jejunum of broiler breeders and offspring; (D) sIgA content in the jejunum of broiler breeders. *P < 0.05, compared with another group. Abbreviations: CD, crypt depth; CLDN-1, claudin-1; CON, control, birds fed a basal diet; OCLN, occludin; SB, sodium butyrate, birds fed a basal diet containing 300 mg/kg SB; sIgA, secretory immunoglobulin A; VH, villi height; ZO-1, zonula occludens 1.

Jejunal and ileal intestinal barrier genes in breeders and offspring were used to determine the effect of SB on gut development and immune function in breeders and their offspring. The developmental morphology of the jejunum and the level of sIgA were also measured (Figure 2). Therefore, SB supplementation upregulates the expression of claudin-1 (***CLDN-1***) in the jejunum of both breeders and offspring. Gene expression of zonula occludens 1 (***ZO-1***), *CLDN-1*, and occludin (***OCLN***) in the ileum of SB breeders was upregulated (*P* < 0.05). A similar increase was observed in the ileum of the offspring. In addition, breeders in the SB group significantly decreased the CD in the jejunum (*P* = 0.04), whereas their offspring had increased VH (*P* = 0.03). The jejunal sIgA level was remarkably upregulated in breeders (*P* < 0.001) but not in offspring.

### Microbial Community

**Figure 3:**
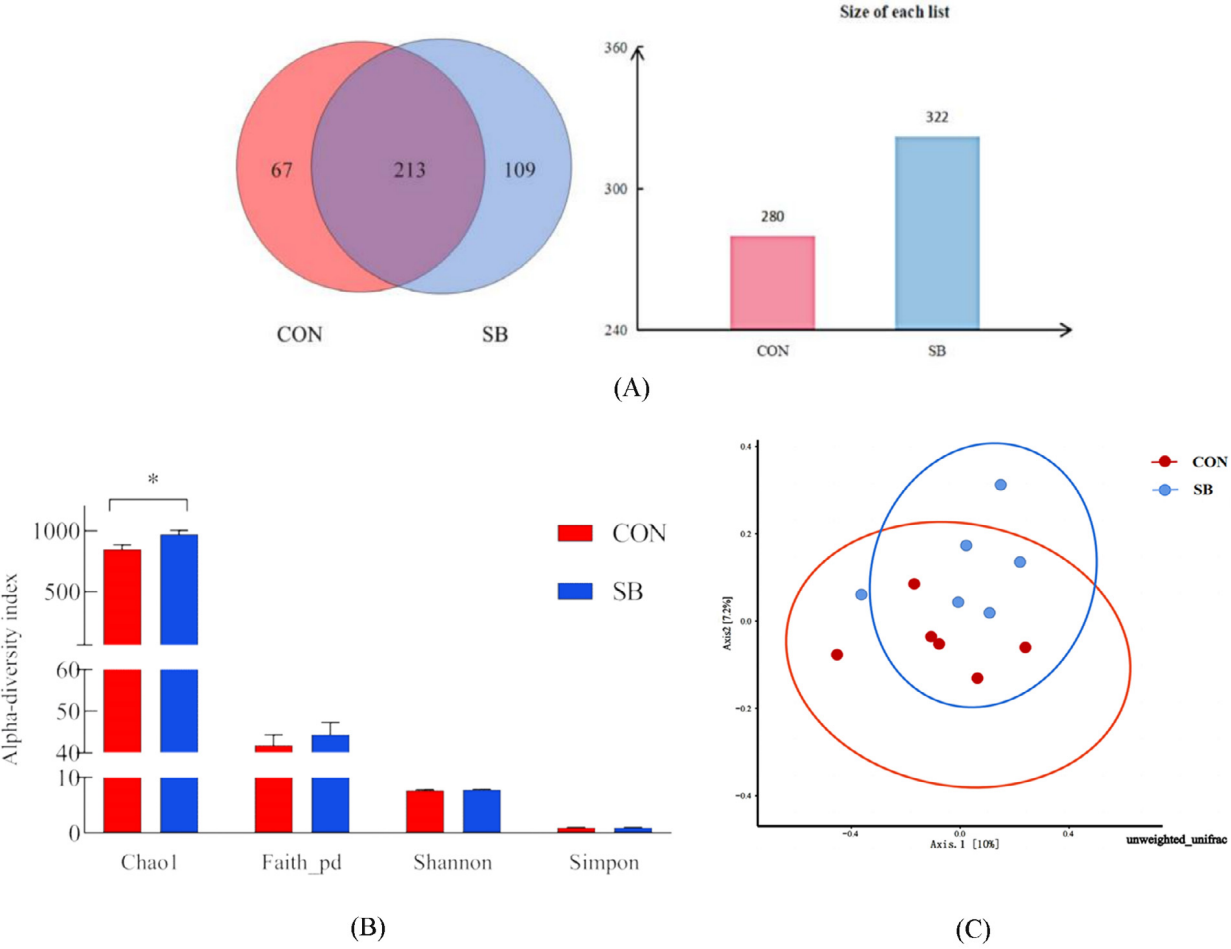
Effects of dietary sodium butyrate on the cecal microbiota diversity of broiler breeders. (A) Venn diagram based on the OTU level; (B) α diversity based on the Chao1, Faith-pd, Shannon, and Simpson indices; (C) PCoA based on unweighted UniFrac distance. *P < 0.05, compared with another group. Abbreviations: CON, control, birds fed a basal diet; SB, sodium butyrate, birds fed a basal diet containing 300 mg/kg SB.

**Figure 4:**
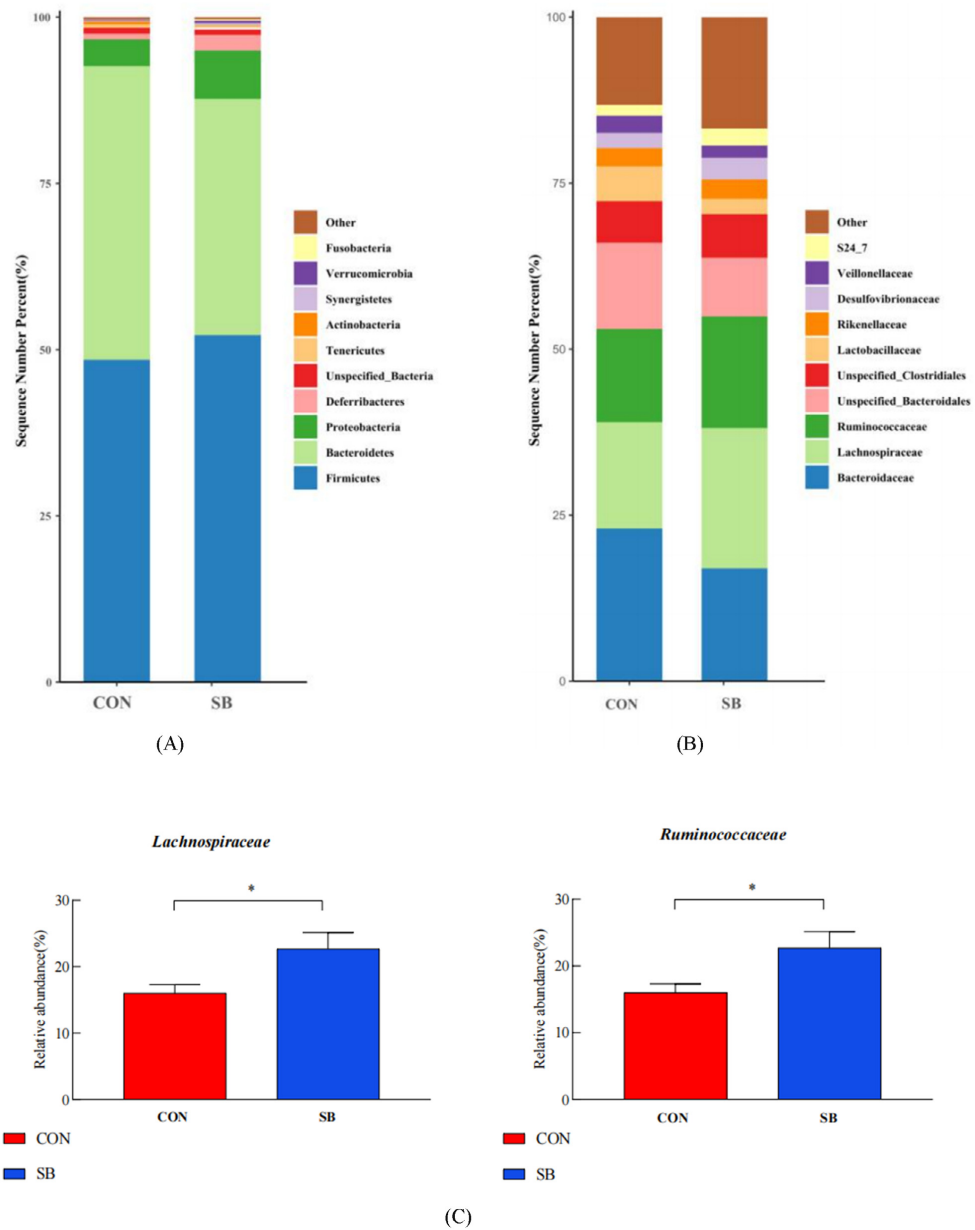
Effects of dietary sodium butyrate on the cecal microbiota composition of broiler breeders. (A) Microbial composition at the phylum level; (B) Microbial composition at the family level; (C) Relative abundances of Lachnospiraceae and Ruminococcaceae at the family level. *P < 0.05, compared with another group. Abbreviations: CON, control, birds fed a basal diet; SB, sodium butyrate, birds fed a basal diet containing 300 mg/kg SB.

The effect of SB on cecal microbes, which are involved in intestinal immunity and barrier function, was investigated via 16s rRNA high-throughput sequencing. According to the Venn diagram (Figure 3A), 54.3% of OTUs were shared between the 2 groups of cecal microbial communities, and a higher number of OTUs was observed in the SB group. Unweighted UniFrac distance PCoA analysis showed differences between the SB treatment and CON group, with a more significant aggregation of cecal microbes in the SB group (Figure 3C). Chao1, Faith-pd, Shannon, and Simpson indices were used to assess the species abundance of individual samples. The SB treatment group showed significant changes in the Chao1 index (Figure 3B). Therefore, SB diet resulted in high microbial diversity in broiler breeders. At the phylum level, the 3-top phyla, *Firmicutes, Bacteroidetes*, and *Proteobacteria*, comprised more than 95% of the total microbial community, while the addition of SB resulted in alterations in the abundance of those microorganisms at the phylum level (Figure 4A). At the family level, the top 3 dominant families were *Bacteroidaceae, Lachnospiraceae*, and *Ruminococcaceaae*, which account for more than 50% of the total microbial community (Figure 4B). The top 10 families at the family level were analyzed, and the results show that SB treatment significantly elevated *Lachnospiraceae* (*P* = 0.004) and *Ruminococcaceae* (*P* = 0.03) (Figure 4C).

## DISCUSSION

Supplementation with SB promotes intestinal development and improves immunity by providing energy to renew intestinal mucosal epithelial cells (Šuligoj et al., 2020). The dissociated butyric acid penetrates freely into the cytoplasm of bacteria to inhibit DNA replication and dissociate the nutrient transport system of bacteria, thus providing SB with a broad-spectrum antibacterial effect (Guilloteau et al., 2010). The positive effects of SB as a feed additive on broiler breeders and offspring, have been proven extensively. Supplementary SB can improve the performance of broilers by improving intestinal development (Liu et al., 2017). Dietary butyrate has a regulatory effect on the transport of mineral elements (Gommers et al., 2022), while SB improves egg production and shell strength and reduces feed conversion (Wang et al., 2021b; Zhang et al., 2022b). Feeding SB for 5 wk improved the immunity and growth potential of 40-wk-old laying hens’ offspring (Gong et al., 2020). Consistent with previous studies, SB improved egg production, the feed conversion ratio and improved egg-shell strength in broiler breeders. The yolk color was ranked significantly lower in the SB group than in the CON group. Egg yolk color depends on the source and level of carotenoids in the feed (Nabi et al., 2020), and the higher egg production rate resulted in a significant decrease in yolk color because of the determined level of carotenoids in the feed as a result of the maternal fixed feed intake. In the poultry industry, the management tends to control fatty acid ratios and reduce cholesterol levels in breeders and laying hens as a nutritional strategy to counteract the negative effects of fat accumulation in late production on performance and health status (Tan et al., 2022). In the present study, SB reduced cholesterol and triglyceride levels in maternal and offspring serum, and this process may be associated with increased liver antioxidant capacity and altered gut microbes (Alves-Bezerra and Cohen 2017; Schoeler and Caesar, 2019). Vitamins A and E are important antioxidants that contribute to the stability of reproductive function in broiler breeders and improve performance and egg quality (Ki-Moon et al., 2010; Abd El-Hack et al., 2019). In addition, vitamin K can increase the quality and thickness of the eggshell and thus improve egg quality (Cornescu et al., 2022). In the present study, these fat-soluble vitamins increased in maternal and offspring serum, and eggs as a result of SB supply, suggesting that more fat-soluble vitamins are depleted in the breeder. This result provides a basis for improved egg quality and reproductive performance.

As poultry age, the antioxidant capacity and immunity of breeder chickens decline rapidly, thus decreasing the reproductive performance and economic efficiency (Amevor et al., 2022). A complex system of antioxidant enzymes is the key to antioxidant damage in poultry. In addition to oxidizing superoxide radicals into hydrogen peroxide and molecular oxygen, superoxide dismutase acts as an antioxidant enzyme (Wang et al., 2021a). It has been shown for instance that SB alleviates oxidative stress caused by a high-fat diet by activating SOD in the rat liver (Sun et al., 2019). SB increases the T-SOD enzyme activity and subsequently increases the germ cell viability in 45-wk-old breeder chickens (Alhaj et al., 2018). In the present study, SB increased T-SOD concentrations in the eggs of broiler breeders and in the liver and serum of offspring, demonstrating that SB improved the antioxidant capacity of broiler breeders and offspring. The process of oxidative stress can be further accelerated by inflammation, which is one of the downstream reactions (Shen et al., 2021). Following immune challenges from the environment in poultry, endotoxins enter the internal environment through the damaged intestinal barrier and stimulate increased secretion of cytokines, such as IL-1β, IL-4, and IL-6 through the *NF-κB* signaling pathway (Kong et al., 2022). Supplementation with SB blocks the attenuation of endotoxin-induced inflammatory responses by reducing cytokine secretion in various inflammatory models (Dang et al., 2021). Immunoglobulins can regulate the specific immunity of animals against entering harmful substances through antibodies, of which IgA and IgG are the main immunoglobulins. Poultry immune function can also be significantly improved via dietary SB by increasing the secretion of immunoglobulins (Ghanim and isihak, 2022). Newly hatched broiler offspring have weak immune functions due to insufficiently developed immune organs, thus rendering them poorly equipped to fight disease. Freshly hatched poultry can achieve innate immunity through maternal antibodies (Berghof et al., 2013). Maternal antibody immunity refers to the transfer of antibodies to the offspring through the yolk sac. IgG and IgA are often used as indicators to evaluate maternal antibodies’ effect on the offspring's immune function (Fan et al., 2018). By contrast, the maternal activation of immune function through exogenous nutrition promotes the immune response in offspring (Verwoolde et al., 2022). In the present study, dietary SB improved the immune function of broiler breeders. An increase in IgA may indicate an improvement in immunity (Sun et al., 2015). The offspring's innate and acquired immune function was significantly enhanced as indicated by the decrease in IL-1β, IL-4, and IL-6, also showed by an increase in immunoglobulins in the serum of the offspring.

Starting from 40 wk of age, the reproductive capacity of broiler breeders remarkably decreases, as reflected by a dramatic loss in egg production rate (Iqbal et al., 2016). The progressive deterioration of intestinal health with age is a key factor in the performance of broiler breeders (Maina et al., 2022). Intestinal integrity, development of the intestinal epithelium, and a well-functioning intestinal microbial community are essential to prevent pathogenic bacteria from infecting poultry (Bindari and Gerber, 2021). Intestinal morphology indicates the ability of the gut to absorb nutrients and its health. However, tight junction proteins can prevent endotoxins from entering the circulation by protecting the intestinal barrier (Di Tommaso et al., 2021). In addition, secretory IgA provides mucosal immunity in intestinal health and strengthens the gut against pathogenic bacteria (Wang et al., 2020), and these findings have been confirmed in the maternal jejunum. Gut health and homeostasis depend on butyrate, the primary food source of colonocytes (Estaki et al., 2016). In vitro assays have shown that of the use of 2 mM sodium butyrate can promote intestinal barrier function in Caco-2 cells by significantly increasing trans-epithelial electrical resistance (Peng et al., 2007). Animal experiments have also shown that dietary butyrate can affect growth performance by improving the structure and function of the tight junction-mediated intestinal epithelial barrier (Song et al., 2017). In the present study, SB improved intestinal development in broiler breeders and offspring, significantly reduced maternal jejunal crypt depth, promoted an increase in offspring villus height, and upregulated *OCLD* gene expression in the jejunum and ileum of broiler breeders and offspring. Therefore, SB could improve intestinal development and intestinal barrier function in broiler breeders and offspring.

Poultry gut microorganisms effectively interact with the active ingredients in the feed, thus playing a regulatory role in nutrition and immunity; for instance, an *E. coli* challenge led to inflammatory responses in broilers and decreased intestinal health (Wu et al., 2021), while the probiotic *Pediococcus acidilactici* promoted intestinal immunity by restoring intestinal barrier function (Jazi et al., 2018). SB enhances intestinal immune function by modulating the community of microorganisms that colonize the intestine (Ma et al., 2020).

Abundant intestinal microbial communities enhance intestinal microecology's ability to maintain stable homeostasis and reduce host susceptibility to pathogens while preventing inflammatory responses (Shi et al., 2017). As a green alternative to antibiotics, SB improves microbial diversity and strengthens intestinal barrier function, leading to a microecological environment that is more tolerant to invading harmful pathogens in the intestine (Zou et al., 2019; Allam-Ndoul et al., 2020). Therefore, improving microbial abundance by dietary SB is associated with strengthening the host's intestinal barrier function.

In vitro and in vivo studies have shown the inhibition of the antibacterial activity of sodium butyrate against deleterious bacteria; for instance, SB inhibited the infection of mammary epithelial cells with *Staphylococcus aureus* (Alva-Murillo et al., 2015). Animal research also revealed that SB reduced the number of *Salmonella* spp. in the cecum of broiler chickens (Liu et al., 2017). Furthermore, SB increased the abundance of *Ruminococcaceae* and *Lachnospiraceae* at the family level in the cecum of broiler breeders. The *Ruminococcaceae* family belongs to the phylum *Firmicutes*, and it plays an important role in the decomposition and utilization of plant fibers. The isolated *Ruminococcaceae* probiotics could alleviate intestinal inflammation by increasing butyrate production. The effective role of various functional additives, such as xylanase, acts by increasing the abundance of *Lachnospiraceae* which in turn leads to the production of butyric acid and other secretions which fulfill additional roles to improve the host's intestinal microbial community (Berger et al., 2021). Butyric acid produced by microbial fermentation acts as an energy source for the intestinal epithelium improving the intestinal barrier function and promoting the abundance of beneficial bacteria, thus promoting intestinal health in broiler breeders. Additional studies support the notion that the gut microbiota influences the absorption and postabsorption metabolism of fat-soluble vitamins in the gut, which is achieved through the secretion of fat-soluble vitamin transporter proteins and the indirect regulation of fat-soluble vitamin receptors through the secretion of the secondary bile acid lithocholic acid (Stacchiotti et al., 2021). A series of SCFA-secreting microorganisms, such as *Ruminococcaceae*, plays a key role (Zhuang et al., 2017), and explains the high amount of fat-soluble vitamins detected in broiler breeders and progeny in this study. Therefore, the change in microbial abundance and community composition possibly resulted from the bidirectional regulation of microbes and nutrients by sodium butyrate supplementation. SB enhanced intestinal barrier function by altering cecum microbial metabolism.

## CONCLUSIONS

In conclusion, dietary SB improved the production and reproductive performance of broiler breeders and ameliorated egg quality by inhibiting inflammation and oxidative stress in intensive rearing conditions. The alteration of the cecal microflora regulated by SB might explain the enhanced intestinal barrier function and absorption of fat-soluble vitamins in broiler breeders. Furthermore, the supplementation of broiler breeder diets with SB promoted both the immune function and intestinal health of the offspring.

## Appendix Supplementary materials

Image, application 1

## Footnotes

Supplementary material associated with this article can be found in the online version at doi:10.1016/j.psj.2023.102658.
