# Supplementary material for: Dietary sodium butyrate improves female broiler breeder performance and offspring immune function by enhancing maternal intestinal barrier and microbiota
Source: Poult Sci. 2023 Mar 18;102(6):102658. doi: 10.1016/j.psj.2023.102658 (PMC10127124; doi:10.1016/j.psj.2023.102658)
Supplement: Supplementary file 1 [file mmc1.doc]

**Supplementary Table S1.** Composition and nutritional levels of the basal diet.

| Item | 40-50 weeks | 51-60 weeks |
| --- | --- | --- |
| Ingredient (%) |  |  |
| Corn | 60.87 | 62.90 |
| Soybean meal (43%) | 20.30 | 18.31 |
| Flour | 5.00 | 5.00 |
| Salt | 0.35 | 0.35 |
| Limestone | 8.00 | 8.00 |
| Dicalcium phosphate | 1.60 | 1.60 |
| Soybean oil | 3.00 | 3.00 |
| Vitamin premix^1^ | 0.025 | 0.025 |
| Mineral premix^2^ | 0.20 | 0.20 |
| Choline chloride (50%) | 0.15 | 0.10 |
| Methionine (99%) | 0.14 | 0.15 |
| Lysine (70%) | 0.07 | 0.07 |
| Threonine (98.5%) | 0.05 | 0.05 |
| Phytase (20000U) | 0.02 | 0.02 |
| Total | 100.00 | 100.00 |
| Nutritional level |  |  |
| Metabolizable energy, kcal/kg | 2850 | 2900 |
| Crude protein | 14.50 | 14.00 |
| Lysine | 0.65 | 0.62 |
| Methionine | 0.57 | 0.53 |
| Calcium | 0.60 | 0.58 |
| Total phosphorus | 0.60 | 0.60 |
| Available phosphorus | 0.40 | 0.40 |

^1^Provided per kilogram of compound diet：vitamin A, 15000 IU; vitamin D3, 4000 IU; vitamin E, 100 mg; VK, 5 mg; vitamin B1, 6 mg; vitamin B2, 15 mg; nicotinic acid, 60mg; pantothenic acid, 20mg; vitamin B6, 4mg; biotin, 0.22 mg; folic acid, 4 mg; vitamin B12, 0.05 mg; I, 1.25 mg.

^2^Provided per kilogram of compound diet: Fe, 20 mg; Mn, 120 mg; Se, 0.3 mg; Zn, 110 mg. Nutrition level was calculated value.

**Supplementary Table S2.** Primers used for real-time qPCR.

| Gene | Accession Number | Primer sequence, 5′→ 3′ | Product size (bp) |
| --- | --- | --- | --- |
| *ZO-1* | XM_015278981.2 | CTTCAGGTGTTTCTCTTCCTCCTCTC | 131 |
|  |  | CTGTGGTTTCATGGCTGGATC |  |
| *OCLN* | NM_205128.1 | TCATCGCCTCCATCGTCTAC | 142 |
|  |  | TCTTACTGCGCGTCTTCTGG |  |
| *CLDN1* | NM_001013611.2 | CTGATTGCTTCCAACCAG | 140 |
|  |  | CAGGTCAAACAGAGGTACAAG |  |
|  |  | ATGTACTGCGGGTTGGTCAT |  |
| *GAPDH* | NM_204305.1 | GCCCAGAACATCATCCCA | 137 |
|  |  | CGGCAGGTCAGGTCAACA |  |
| *β-actin* | L08165 | CAGACATCAGGGTGTGATGG | 183 |
|  |  | TCAGGGGCTACTCTCAGCTC |  |

*ZO-1* = zonula occludens 1; *OCLN* = occludin; *CLDN1* = claudin-1; *GAPDH* = glyceraldehyde-3-phosphate dehydrogenase.
